# Causes of neonatal mortality using verbal autopsies in rural Southern Nepal, 2010–2017

**DOI:** 10.1371/journal.pgph.0001072

**Published:** 2022-09-15

**Authors:** Ayesha R. Saya, Joanne Katz, Subarna K. Khatry, James M. Tielsch, Steven C. LeClerq, Luke C. Mullany

**Affiliations:** 1 Neonatal-Perinatal Medicine, Johns Hopkins University, Baltimore, MD, United States of America; 2 International Health, Johns Hopkins University Bloomberg School of Public Health, Baltimore, MD, United States of America; 3 Nepal Nutrition Intervention Project-Sarlahi, Lalitpur, Bagmati, Nepal; 4 Department of Global Health, Milken Institute School of Public Health, George Washington University, Washington, DC, United States of America; University of Malaya Faculty of Medicine, MALAYSIA

## Abstract

The burden of neonatal mortality remains high worldwide, particularly in South Asia. Verbal Autopsy is a method used to identify cause of death (COD) where vital registration capabilities are lacking. This study examines the causes of neonatal mortality in a large study population in rural Southern Nepal. The data used is from a larger cluster-randomized community-based trial. The study includes 984 neonatal deaths with complete verbal autopsy information which occurred between 2010 and 2017. The InterVA-5 software was used to identify COD. COD included severe infection (sepsis, pneumonia, meningitis/encephalitis), intrapartum related events (identified as birth asphyxia), congenital malformations, and other. The neonatal mortality rate was 31.2 neonatal deaths per 1000 live births. The causes of neonatal mortality were identified as prematurity (40%), intrapartum related events (35%), severe infection (19%), congenital abnormalities (4%), and other (2%). A high proportion, 42.5% of neonatal deaths occurred in the first 24 hours after birth. Over half (56.4%) of deaths occurred at home. This large prospective study identifies population level neonatal causes of death in rural Southern Nepal, which can contribute to national and regional COD estimates. Interventions to decrease neonatal mortality should focus on preventative measures and ensuring the delivery of high risk infants at a healthcare facility in the presence of a skilled birth attendant.

## Introduction

The global burden of neonatal mortality remains high. In 2019, 2.4 million deaths occurred in the neonatal period, comprising 46% of all deaths under 5 years of age. Of these neonatal deaths, 1 million occur within the first day after birth [1]. Since the progress in decreasing neonatal mortality worldwide has lagged behind the decrease in under-5-mortality [2], it is imperative to understand the causes of neonatal mortality in order to establish targeted public health interventions.

Worldwide, the highest burden of neonatal deaths is in sub-Saharan Africa and South Asia. In 2010, the national neonatal mortality rate (NMR) in Nepal was 26.5 neonatal deaths per 1000 live births as compared to a worldwide NMR of 22.1 neonatal deaths per 1000 live births. This has decreased over time to 16.9 neonatal deaths per 1000 live births in Nepal and 17 neonatal deaths per 1000 live births worldwide in 2020 [3]. This decline in the NMR has coincided with a decrease in the total fertility rate, improvements in education, increased skilled birth attendance and antenatal care visits, and an increased coverage of community based child interventions [4]. The three main causes of neonatal mortality in South Asia from 2012–2016 were perinatal asphyxia (40%), severe neonatal infections (35%), and preterm birth (19%) [5].

Vital registration systems, which count births and deaths, are lacking in many parts of the world. Only 26% of the world’s population live in countries with complete registration of deaths, and this proportion is especially low in Africa (7% overall and less than 3% in sub-Saharan Africa) [6]. It is particularly difficult to accurately count neonatal deaths in resource-limited settings because many neonatal deaths occur early and at home and therefore go unrecorded. Additionally, neonatal signs and symptoms are often nonspecific making the accurate identification of cause of death (COD) difficult.

Verbal Autopsy (VA) is a method used to ascertain the cause of a death based on an interview with caregivers that can be applied for deaths without certification of medical causes. Initial development of VA occurred in the 1950s and was standardized by the WHO in 2007 [7]. With widespread use, it allows for the determination of population level COD estimates in low- and middle-income countries where adequate civil registration systems are lacking.

This study uses verbal autopsy to identify the causes of neonatal deaths from 2010 to 2017 in the Sarlahi District of Nepal using VA methods.

## Methods

### Ethics statement

Verbal consent was obtained by project workers using an oral script. The study was approved by the Ethical Review Committee of the Institute of Medicine, Tribhuvan University (Kathmandu, Nepal) and the Institutional Review Board of the Johns Hopkins Bloomberg School of Public Health (Baltimore, MD).

### Study design

This observational study was nested within a larger cluster-randomized community-based trial studying the impact of topical skin emollient application on newborn mortality and morbidity (ClinicalTrials.gov, NCT01177111) [8]. The trial was performed from 2010 to 2017.

Communities (clusters) were randomized to promotion of topical applications to newborn skin (“newborn massage”) with sunflower seed oil (intervention) or the traditional mustard seed oil (comparison), which is used almost universally as a standard neonatal care practice [9].

Pregnant women were identified through regular monitoring by local female project workers; married women aged 15–35 were visited at home every five weeks, queried about menstruation, and offered pregnancy tests. Once they were confirmed to be pregnant, they were enrolled in the trial, date of last menstrual period (LMP) was recorded, and they were followed every month during pregnancy. They were randomized to receive either sunflower seed oil or mustard seed oil. During the enrollment and follow up visits, women across both groups were provided a common set of basic services to meet recommendations for essential newborn care as part of the standard Nepal Neonatal Health Strategy [10]. These included a clean delivery kit, provision of iron folate and deworming, chlorhexidine cleansing of the cord, basic educational message on antenatal and essential newborn care (maternal nutrition during pregnancy, danger signs and associated care-seeking, early/exclusive breastfeeding, clean/hygienic delivery, cord care, hand-washing, and thermal care).

### Study site

This study was performed in the Sarlahi district, a rural area in Southern Nepal. Antenatal care in this setting is limited and does not include the administration of antenatal corticosteroids for fetal lung maturation.

### Sampling

This study included neonatal deaths which occurred in the study population between 2010 and 2017.

### Data collection

An attempt was made to visit the neonate as soon after birth as possible (day 0) to weigh the neonate and collect information about labor and delivery. After birth, a data collector visited the home on days 1, 3, 7, 10, 14, 21, and 28. At each visit after birth, the neonate was examined for vital status and signs of illness, and the mother/caretaker reported signs of morbidity, care-seeking, and newborn care practices. If the neonate died, a verbal autopsy was performed.

The verbal autopsy survey used in the study (S1 Text) was based on the 2007 WHO verbal autopsy instrument [7]. The components of the survey included location and time of death, care seeking practices, if any, prior to death, and the ascertainment of characteristics of the neonate (presence of congenital abnormality, perceived size of the neonate). The survey also assessed the presence of 26 symptoms prior to death of the neonate. It ended with an opportunity for a description, in the respondent’s own words, of how the neonate died.

The verbal autopsies were conducted by local supervisory staff who did not have medical training but had significant training by a physician in the conduct of the verbal autopsies. These staff have years of experience conducting verbal autopsies in prior studies in this district. The interviews were conducted in Nepali (the national language) or Maithili (a local language), depending on the comfort level and choice of the family being interviewed. The timing of the verbal autopsy was intended to be as soon after death as possible, given mourning practices and taking into account the family’s wishes. The median time between a neonatal death and performance of the verbal autopsy was 7 days.

### Data analysis

There are three automated models which are used to interpret the data from the verbal autopsy instrument, namely InterVA, InSilicoVA, and Tariff [11–13]. The survey responses were interpreted using InterVA-5, which uses a Bayesian mathematical model (developed to align with the 2016 WHO VA instrument as well as prior versions [11] to identify COD [14]. InterVA-5 lists up to 3 likely causes of death for each case. For this study, the first cause listed with the highest likelihood is designated as the cause of death. The identified causes of death as determined by InterVA5 of sepsis, pneumonia, meningitis and encephalitis are grouped together as “severe infection” for the purposes of this study. We used R statistical software and Microsoft Excel to perform descriptive statistics.

Neonates with gestational ages less than 37 weeks were classified as premature. Miscarriage was defined as loss of a fetus before 28 weeks gestation [15]. Stillbirths were defined as neonates who did not breathe, cry, or move after birth at or after 28 weeks gestation [16]. Gestational age (GA) was based on time elapsed from LMP (self-reported) to date of birth. Neonatal deaths with complete verbal autopsy information were included. Neonates who were live births but with GAs less than 22 weeks or greater than 44 weeks were excluded from analyses involving GA.

## Results

The NOMS study followed 34,541 pregnancies from 2010 to 2017. Miscarriages occurred in 4.8% of pregnancies, and the stillbirth rate was 26.1 stillbirths per 1000 pregnancies. Of the 32,050 live births followed, there were 1001 deaths which occurred in the neonatal period, resulting in a neonatal mortality rate (NMR) of 31.2 neonatal deaths per 1000 live births. The NMR of the study population decreased over time from 34.7 deaths per 1000 live births in 2011 to 27.5 deaths per 1000 live births in 2016. The national NMR was 21.7 deaths per 1000 live births in 2016 [17] (S1 Fig).

Data collectors visited the homes of infants as soon after birth as possible and attempted to revisit the homes at predetermined intervals. 53.0% of verbal autopsies occurred within 7 days after the infant’s death, 21.4% occurred between 7 and 28 days after death, and 25.5% occurred after 28 days after death. 0.6% of live born neonates had an estimated GA less than 22 weeks and 4.0% of live born neonates had an estimated GA greater than 44 weeks and were excluded from analyses involving GA.

Throughout the study period, most deliveries and deaths occurred at home. Neonatal births at home decreased over the study period (from 71% in 2011 to 47% in 2017, p<0.05) and the proportion of deliveries occurring at healthcare facilities (hospitals and clinics) increased. Infants died less frequently at home during the study period (67% in 2011 to 50% in 2017, p<0.05). Notably, 11% of all neonatal deaths occurred on the way to a healthcare facility (S2 Fig).

The 984 neonatal deaths with complete verbal autopsy information were analyzed. 42.5% of the 984 neonatal deaths occurred within 24 hours after birth, and of those who died within 24 hours after birth, 41.4% of the deaths occurred within the first hour after birth. Neonatal deaths were concentrated in the first hour of the first day after birth (S3 Fig).

Among the 984 neonatal deaths, 44.2% of neonates were born preterm. A higher proportion of neonates that died within 24 hours after birth were preterm as compared to those who died in the late neonatal period, 7–28 days (50.3% versus 38.9%, respectively, p<0.05). Additionally, a higher proportion of preterm infants born less than 34 weeks died within the first day after birth as compared to late preterm infants (53.5% versus 37.1%, p<0.05). There were slightly more males than females who died in the neonatal period (54.6% versus 45.0%, respectively, p<0.05) but there were no appreciable differences by the time of death. A substantial percentage of neonatal deaths, 10.8%, occurred among twin and triplet infants (S1 Table).

**Fig 1:**
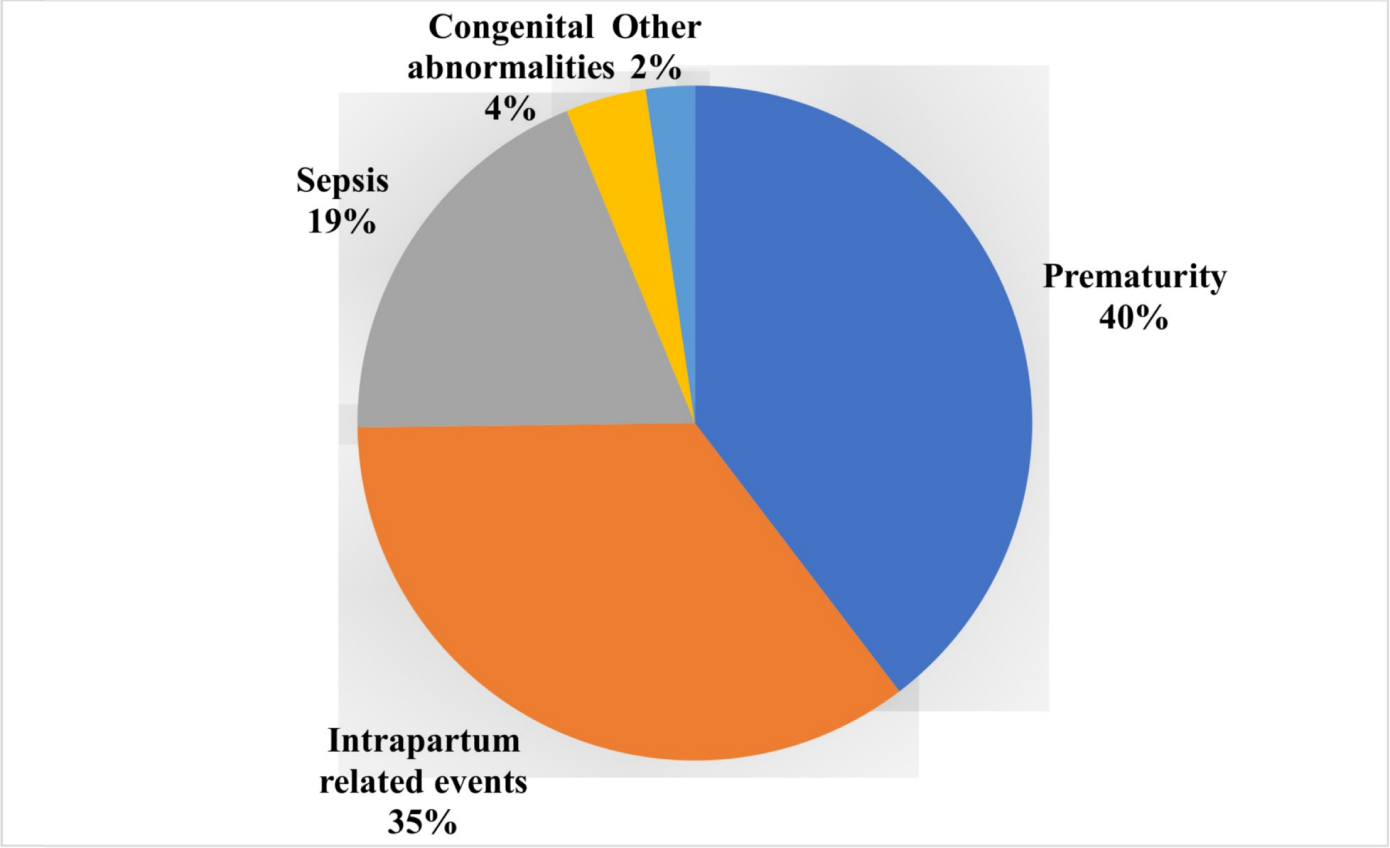
Causes of neonatal mortality in the study area, Sarlahi District from 2010 to 2017 using the InterVA-5 algorithm.

Fig 1 shows the causes of neonatal deaths as assigned by the InterVA-5 software. 35% of neonatal deaths were due to intrapartum related events, 40% were due to prematurity, 19% due to severe infection, 4% due to congenital abnormalities, and 2% were assigned other as a cause. Among the 414 deaths in preterm infants (44.2% of deaths with included GAs), 278 (67.1%) were assigned prematurity as a COD. Of the remaining neonatal deaths among preterm infants, 73 were determined to be due to intrapartum related events (17.6%), 40 due to severe infection (9.7%), 14 due to congenital malformation (3.4%), and 9 due to other causes (2.2%). The average GA of infants who were determined to die of prematurity was 34.0 ± 4.7, and of the 379 deaths with included GAs due to prematurity, 278 infants, or 73.4%, were preterm. A higher proportion of preterm neonates with gestational ages less than 34 weeks were assigned prematurity as a COD as compared to late preterm infants born 34 to 37 weeks gestation (72.6% versus 56.4%, p<0.05). Of the 102 infants assigned prematurity as a COD whose GA was greater than or equal to 37, 77 infants (75.5%) were thought to be of average size (per caregiver report). The average GA among twins and triplets who died in the neonatal period was 33.2 ± 3.2, and a higher proportion of deaths from prematurity (21.0%) were among twins and triplets as compared to other causes of death. Of the 346 neonatal deaths due to intrapartum related events, 50.9% occurred within 24 hours after birth, 40.5% occurred between 1 and 7 days after birth, and 8.7% occurred between 7 and 28 days after birth. Of the 187 neonatal deaths due to severe infection, 3.2% occurred within 24 hours after birth, 41.2% occurred between 1 and 7 days after birth, and 55.6% occurred between 7 and 28 days after birth (Table 1). There were no appreciable differences in COD between males or females (Table 1) or between infants that were exposed to mustard seed oil or sunflower seed oil. Most deaths, regardless of the cause, occurred at home. The highest proportion of deaths that occurred in hospitals, 34.6%, occurred among infants who died due to prematurity (Table 1).

**Table 1:** Demographic and clinical characteristics for each cause of neonatal deaths in Sarlahi, Nepal, 2010–2017.

	Total	Prematurity	Intrapartum related events	Severe Infection	Congenital Malformation	Other
n	984	390 (39.6)	346 (35.2)	187 (19.0)	38 (3.9)	23 (2.3)
**Premature**						
Preterm <34w0d	274	199 (72.6)	41 (15.0)	19 (6.9)	10 (3.6)	5 (1.8)
Preterm 34w0d- 36w6d	140	79 (56.4)	32 (22.9)	21 (15.0)	4 (2.9)	4 (2.9)
**Sex (n, %)**						
Male	537	233 (43.4)	176 (32.8)	100 (18.6)	16 (3.0)	12 (2.2)
Female	447	157 (35.1)	170 (38.0)	87 (19.4)	22 (4.9)	11 (2.5)
**Age at Death (n, %)**						
Death <24 hours after birth	418	214 (51.2)	176 (42.1)	6 (1.4)	10 (2.4)	12 (2.9)
Death 1–7 days after birth	380	140 (36.8)	140 (36.8)	77 (20.3)	17 (4.5)	6 (1.6)
Death 7–28 days after birth	186	36 (19.4)	30 (16.1)	104 (55.9)	11 (5.9)	5 (2.7)
**Multiples (n, %)**						
Singleton	878	308 (35.1)	335 (38.2)	180 (20.5)	34 (3.9)	21 (2.4)
Twin	99	76 (76.8)	11 (11.1)	7 (7.1)	4 (4.4)	1 (1.0)
Triplet	7	6 (85.7)	0 (0)	0 (0)	0 (0)	1 (14.3)
**Location of Death (n, %)**						
Home/maiti	555	181 (32.6)	208 (37.5)	126 (22.7)	27 (4.9)	13 (2.3)
Hospital	227	135 (59.5)	55 (24.2)	23 (10.1)	6 (2.6)	8 (3.5)
Other health facility	74	46 (62.2)	21 (28.4)	5 (6.8)	1 (1.4)	1 (1.4)
In transit to facility	109	22 (20.2)	56 (51.4)	26 (23.9)	4 (3.7)	1 (0.9)
Other	19	6 (31.6)	6 (31.6)	7 (36.8)	0 (0)	0 (0)
**Location of Birth (n, %)**						
Home/maiti	436	139 (31.9)	167 (38.3)	97 (22.2)	24 (5.5)	7 (1.6)
Hospital	153	73 (47.7)	53 (34.6)	21 (13.7)	3 (2.0)	2 (1.3)
Other health facility	251	97 (38.6)	88 (35.1)	53 (21.1)	9 (3.6)	4 (1.6)
In transit to facility	40	21 (52.5)	11 (27.5)	4 (10.0)	2 (5.0)	2 (5.0)
Other	104	60 (57.7)	27 (26.0)	12 (11.5)	0 (0)	8 (7.7)

**Fig 2:**
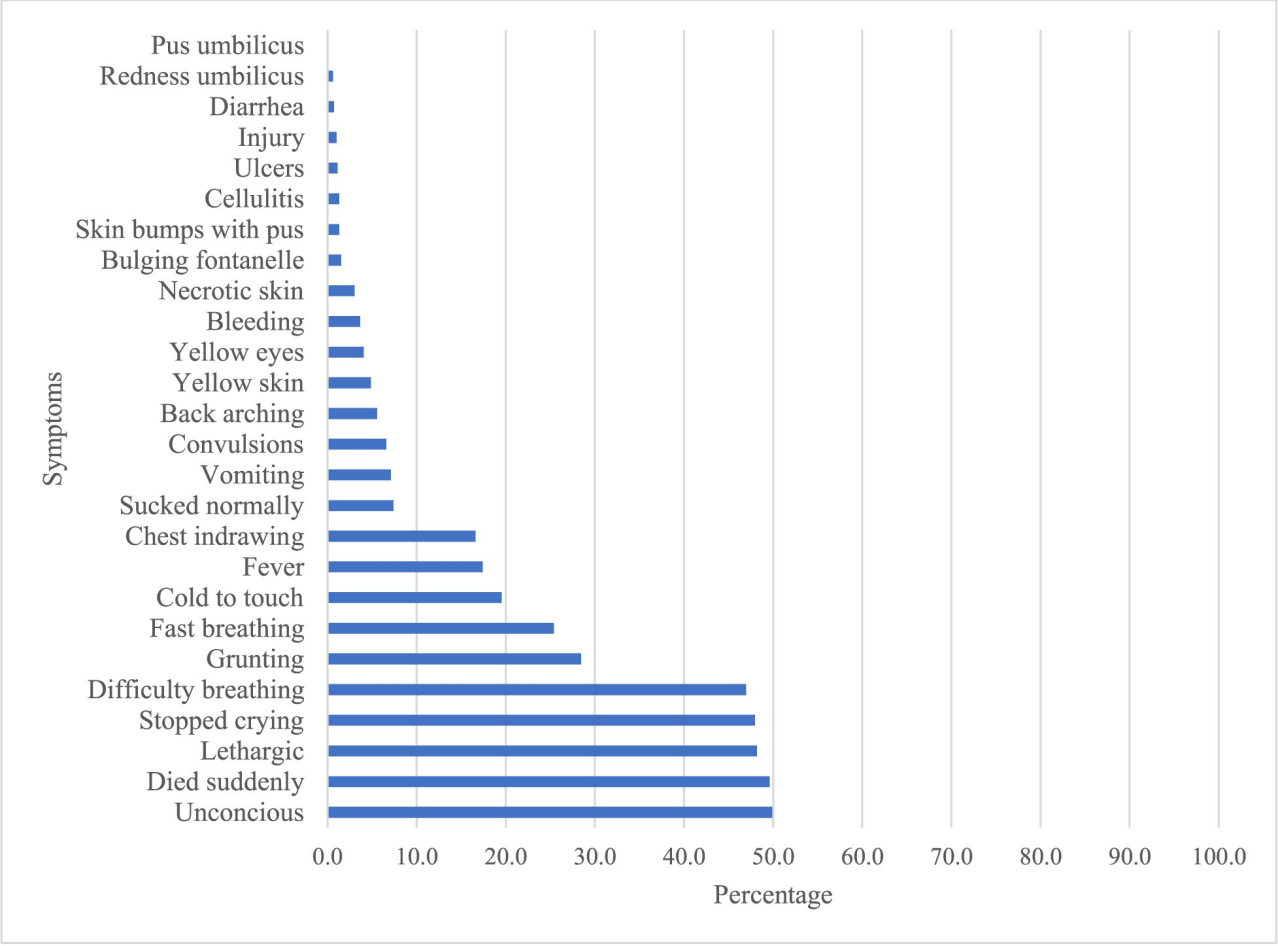
Symptoms reported in verbal autopsy among neonatal deaths (n = 984).

The most commonly reported symptoms in neonatal verbal autopsies were neurologic and respiratory in nature (unconscious, lethargic, stopped crying, difficulty breathing, grunting). The least commonly reported symptoms were related to specific illnesses, such as redness/pus from the umbilicus, diarrhea, or bulging fontanelle (Fig 2). Fever, convulsions, yellow skin, and yellow eyes were more commonly reported in infants who died in the late neonatal period (S4 Fig). The VA questionnaire includes the question “Did the infant appear healthy and die suddenly?”. Of the 984 deaths, 490 (49.8%) of respondents answered “yes” to this question. Of those 490 respondents who answered yes, 392 (80%) of respondents reported the presence of at least 1 symptom prior to death.

## Discussion

The accurate estimation of causes of neonatal mortality in low- and middle-income countries is a key priority to guide targeted interventions to reduce neonatal mortality. This is difficult in resource poor settings where the burden of neonatal mortality is high, and many deaths occur out of the healthcare setting. Verbal autopsy is a way to gather information about an infant’s death after it has occurred. Although previously physicians have been used to assign individual causes of death using VA, the process is costly and not consistent between practitioners [18]. Verbal autopsy algorithms exist to provide population level COD information in a cost-effective, reproducible manner.

Using verbal autopsy methods, this analysis uses data from a large population-based study to describe neonatal COD in the Sarlahi District of rural Southern Nepal. The NMR in the study population was 31.2 deaths per 1000 live births, consistently higher than the national NMR during the study period. Most deaths occurred early in the neonatal period, concentrated in the first hour after birth. A higher percentage of neonatal deaths occurred among twin and triplet neonates, and of those born before 34 weeks gestation, a higher percentage died within the first day after birth as compared to late preterm infants born 34–37 weeks gestation. The higher NMR in the Sarlahi district likely reflects the resource limitations in this rural area. The main causes of neonatal mortality in this population were prematurity (40%), intrapartum related events (35%), severe infection (19%), and congenital abnormalities (4%). There was a very small proportion of deaths which had a designated cause of other (2%). This reflects the most to least common causes of neonatal mortality in 2017 in Nepal as determined by the WHO and Maternal and Child Epidemiology Estimation Group (MCEE) [19]. This is in contrast to the study results of Erchick et al., which examined causes of neonatal deaths across six districts in Nepal from 2012–2013 [20]. Among the 338 neonatal deaths, sepsis was the leading cause of death (47%), followed by birth asphyxia (16.6%) and prematurity (13.3%). There are multiple reasons for these differing results. The study by Erchick et al. analyzed deaths from selected geographic areas in Nepal. These areas were quite diverse, and included mountainous, hilly and plains area, whereas our study included only plains. Their areas included locations with likely poorer access to care due to the terrain in the hills and mountains. Nepal is also ethnically quite diverse and their study included more ethnic diversity than ours. This may have affected care seeking behavior. However, the biggest difference likely stems from their use of physicians to assign causes of death compared to the use of an algorithm. Physicians bring a more holistic approach to the assignment of cause of death, but have interobserver reliability issues that are removed by the use of an algorithm. Algorithms are reproducible by definition but are not necessarily more valid [20]. Of infants born preterm <37 weeks gestation, 2/3 were assigned prematurity as a COD. This implies that the InterVA-5 algorithm does not assign prematurity as a cause of death for all preterm infants, and that other, more proximal, COD are often assigned to preterm infants. Prematurity as a proximal COD occurs more often in preterm infants less than 34 weeks gestation as compared to neonates born 34–37 weeks gestation. Of the 379 infants (excluding GA under 22 weeks and greater than or equal to 44 weeks) whose COD was determined to be prematurity, only 278 infants, or 73.4%, were in fact preterm. Of the term infants assigned prematurity as a COD, 75.5% were thought to be of average size according to the caregiver, and 20.6% were thought to be smaller than usual. Since the details of the InterVA-5 algorithm code are not readily available, it is unclear why some infants who were not preterm were assigned prematurity as the cause of death.

Although the proportion of births and deaths that occurred at home decreased over time in this study, almost half of births and deaths occurred at home, often shortly after birth.

These findings suggest that interventions to decrease neonatal mortality should focus on strengthening health systems to provide timely care to vulnerable infants, such as those who are preterm, twins, and/or triplets. Since most neonatal deaths occur early and at home, efforts should be made to provide antenatal care to prevent birth complications and ensure delivery at a healthcare facility in the presence of a skilled birth attendant.

### Strengths

The strengths of this study include the large number of pregnancies enrolled and the comprehensive information obtained on a large number of neonatal deaths. As compared to when deaths might be recorded using another method, the verbal autopsy survey was performed sooner after death allowing for better recall and more reliable information. There was low loss to follow up and information was lacking for only 1.7% of neonatal deaths. The study results are generalizable to similar rural settings in northern India, Bangladesh and Pakistan, which have some of the highest burdens of global neonatal mortality.

### Limitations

The limitations of the study are largely attributed to the inherent weaknesses of using verbal autopsy methods to determine cause of neonatal deaths. It is based on self-reported data which is subject to recall bias. The COD determination is dependent on the Inter-VA5 algorithm, which is meant for COD on the population level and is subject to its own limitations. Additionally, grief may have affected the answering of the surveys.GA in this study was estimated based on maternal report of her LMP, but the range of GAs among live born infants who subsequently died was 7–55 weeks, with the outliers being biologically implausible. There was likely a component of misclassification of some early neonatal deaths as stillbirths, which has previously been well described [21]. Infants, particularly those who are premature and/or those who are not resuscitated at birth, may not breathe, move, or cry at birth, which is the working definition of a stillbirth in this setting. However, infants identified as stillbirths may be individuals who are born alive (with a heartbeat) and may have survived if resuscitative measures were initiated.

A potential limitation of this study is that since data collectors could refer infants for care to the local health post during their frequent home visits, this may have modified the neonatal mortality in this population. However, in this setting, many infants do not receive timely care despite referrals.

This study was done in a rural setting, and therefore has limited external validity to determine COD in nationwide estimates of neonatal mortality.

### Recommendations

Some questions in the VA instrument do not appear to adequately gather the information intended. Half of respondents answered yes to the question “Did the infant appear healthy and die suddenly?” despite most of these respondents reporting other symptoms prior to death.

Although VA methods may not accurately identify individual causes of death, it provides a standardized way to estimate population level causes of death which is crucial to public health programming to prioritize interventions and measure impact.

These results highlight characteristics of neonatal deaths in this population, which occurred early, at home, and more commonly among preterm neonates and those part of a multiple gestation pregnancy. Accordingly, public health efforts should aim to mitigate the burden of neonatal mortality, with an emphasis on these populations.

## Conclusion

This large study identifies the population level causes of neonatal mortality using verbal autopsy methods in a rural district of Southern Nepal, which can inform regional estimates. Most of these deaths occur early, at home, and are due to prematurity, severe infections, and intrapartum related events. As such, interventions to decrease neonatal mortality should focus on preventative measures and ensuring the delivery of high risk neonates at a healthcare facility in the presence of a skilled birth attendant, particularly for neonates that are part of a multiple gestation pregnancy or those who are preterm. Efforts to improve the individual level data obtained on neonatal deaths should be made to inform regional, national, and international estimates.

## Supporting information

S1 Text Verbal autopsy survey. (PDF)

S2 Text PLOS questionnaire on inclusivity in global research. (DOCX)

S1 Fig Neonatal mortality rates from 2011 to 2016 in Nepal (The World Bank data [18]) and in the Sarlahi District (study data). (DOCX)

S2 Fig Location of births (a, n = 28,659) and neonatal deaths (b, n = 984) from 2011 to 2017. (DOCX)

S3 Fig Frequency of Neonatal Deaths in days (a) and within the first day in hours (b).
(DOCX)

S4 Fig Symptoms reported in verbal autopsies by age at death. (DOCX)

S1 Table Demographic and clinical characteristics of neonatal deaths in Sarlahi, Nepal, 2010–2017. (DOCX)
