# Supplementary material for: Causes of neonatal mortality using verbal autopsies in rural Southern Nepal, 2010–2017
Source: PLOS Glob Public Health. 2022 Sep 15;2(9):e0001072. doi: 10.1371/journal.pgph.0001072 (PMC10021801; doi:10.1371/journal.pgph.0001072)
Supplement: S1 Text — (PDF) [file pgph.0001072.s001.pdf]

NEPAL Oil Massage Study – NEONATAL VERBAL AUTOPSY FORM

Week ☐☐☐ **Date** ☐☐-☐☐-☐☐ **Worker ID** ☐☐☐ ______

VDC ☐☐ **Ward** ☐ **Sector** ☐ **HH** ☐☐☐

	NNIPSNUM	First Names	Last Name
**Mother**	☐☐☐☐☐☐	____________	____________
**Father**	☐☐☐☐☐☐	____________	____________
**Child**	☐ of ☐	____________	____________

☐ **Form Status:**

1=completed Now: Met Status:
2=Not met within 6 months (STOP)
6=refused - **STOP**
7=Permanently Moved - **STOP** 1=yes

Vital Status

1=alive

Section A: General Information

**1.**	Relationship of primary respondent to deceased	1=Mother<br>2=Father<br>3=Grandmother<br>4=Grandfather<br>5=Aunt<br>6=Other family member<br>7=TBA/ANM<br>8=Other ____________<br>9=Don't Know	☐
**2.**	Was primary respondent present during the fatal illness or death?	0=No to either<br>1=Yes to illness only<br>2=Yes to death only<br>3=Yes to both<br>9=Don't know	☐
**3.**	Age of primary respondent	15-97 = Age in years<br>98=98 or more<br>99= Don't Know	☐☐
**4.**	Primary respondent years of schooling	00=None<br>01-20 = Years of schooling<br>99=Don't Know	☐☐

Section B: Vital Information

**5.**	Date of Birth<br>(record dd / mm / yy)	☐☐-☐☐-☐☐	
**6.**	Date of Death<br>(record dd / mm / yy)	☐☐-☐☐-☐☐	
**7.**	Time of Death<br>(record hh:mm)	☐☐ : ☐☐ ☐ → 1= 12:00 AM – 11:59 NOON<br>2= 12:00 PM – 11:59 MIDNIGHT	
**8.**	Where did the child die?	1=Home/maiti/family member's house<br>2=Hospital (Go to Q8a)<br>3=Other health facility (Go to Q8a)<br>4=in transit to facility<br>5=Other _______________	☐
	**8a.** At what facility did he/she die?	____	
**9.**	Is the mother still alive?	0=No (Go to Q9a)<br>1=Yes (Go to Q10)<br>9=Don't Know	☐
	**9a.** Did the mother die during or after delivery?	1=During delivery (Go to Q10)<br>2=After delivery (Go to Q9b)<br>9=Don't Know (Go to Q10)	☐
	**9b.** How long after the delivery did she die?	00-90 days<br>91=more than 90 days<br>99=Don't know	☐☐
**10.**	At birth, what was the size of the baby?	1=Very small<br>2=Smaller than usual<br>3=About average<br>4=Larger than average<br>9=Don't Know	☐

Section C: Neonatal Module

**11.**	Did the child have an accident or injury just before death?	0=No (Go to Q12)<br>1=Yes (Go to Q11a)<br>9=Don't Know	☐
	**11a.** What kind of injury or accident?	1=Fall or blow<br>2=Burn<br>3=Severe cut<br>4=Snake bite<br>5=Animal bite<br>6=Road accident<br>7=Other _______________<br>9=Don't Know	☐

	**11b.** How many days after the accident did the child die?	00=Same day<br>01-28 = Number of days<br>99=Don't Know	☐☐
**12.**	Did the baby stop being able to cry?	0=No<br>1=Yes (Go to 12a)<br>9=Don't Know	☐
	**12a.** How long before the baby died did the baby stop crying?	1=Less than 1 day<br>2=One day or more<br>9=Don't know	☐
**13.**	Was the baby able to suckle in a normal way during the first day of life?	0=No (Go to Q13a)<br>1=Yes (Go to Q13b)<br>9=Don't Know (Go to Q13a)	☐
	**13a.** Did the baby ever suckle in a normal way?	0=No (Go to Q14)<br>1=Yes (Go to Q13b)<br>9=Don't Know (Go to Q14)	☐
	**13b.** Did the baby stop being able to suckle in a normal way?	0=No (Go to Q14)<br>1=Yes (Go to Q13c)<br>9=Don't Know (Go to Q14)	☐
	**13c.** How long after birth the baby stop suckling?	00=Less than 1 day<br>01-28 days<br>99=Don't Know	☐☐
	**13d.** Was the baby able to open his/her mouth at the time he/she stopped sucking?	0=No<br>1=Yes<br>9=Don't Know	☐
**14.**	During the illness that led to death did the baby have difficulty breathing?	0=No (Go to Q15)<br>1=Yes (Go to Q14a-b)<br>9=Don't Know (Go to Q15)	☐
	**14a.** At what age did the difficult breathing start	00=Less than 1 day<br>01-28 days<br>99=Don't Know	☐☐
	**14b.** For how many days did the difficult breathing last?	00=Less than 1 day<br>01-28 days<br>99=Don't Know	☐☐
**15.**	During the illness that led to death did the baby have fast breathing?	0=No (Go to Q16)<br>1=Yes (Go to Q15a-b)<br>9=Don't Know (Go to Q16)	☐
	**15a.** At what age did the fast breathing start	00=Less than 1 day<br>01-28 days<br>99=Don't Know	☐☐

	**15b.** For how many days did the fast breathing last?	00=Less than 1 day<br>01-28 days<br>99=Don't Know	☐☐
**16.**	During the illness that led to death did the baby have indrawing of the chest?	0=No<br>1=Yes<br>9=Don't Know	☐
**17.**	During the illness that led to death, did the baby have grunting?	0=No<br>1=Yes<br>9=Don't Know	☐
**18.**	During the illness that led to death, did the baby have spasms or convulsions?	0=No<br>1=Yes<br>9=Don't Know	☐
**19.**	During the illness that led to death, did the baby have any stiffening of the body or arching of the back?	0=No<br>1=Yes<br>9=Don't Know	☐
**20.**	During the illness that led to death, did the baby have fever?	0=No<br>1=Yes (Go to Q20a-b)<br>9=Don't Know	☐
	**20a.** At what age did the fever start?	00=Less than 1 day<br>01-28 days<br>99=Don't Know	☐☐
	**20b.** For how many days did the fever last?	00=Less than 1 day<br>01-28 days<br>99=Don't Know	☐☐
**21.**	During the illness that led to death, did the baby become cold to touch?	0=No (Go to Q22)<br>1=Yes (Go to Q21a)<br>9=Don't Know (Go to Q22)	☐
	**21a.** At what age did the baby start feeling cold to touch?	00=Less than 1 day<br>01-28 days<br>99=Don't Know	☐☐
**22.**	During the illness that led to death, did the baby become lethargic after a period of normal activity?	0=No<br>1=Yes<br>9=Don't Know	☐
**23.**	During the illness that led to death, did the baby become unresponsive or unconscious?	0=No<br>1=Yes<br>9=Don't Know	☐
**24.**	During the illness that led to death, did the baby have a bulging fontanelle?	0=No<br>1=Yes<br>9=Don't Know	☐

**25.**	During the illness that led to death did the baby have pus drainage from the umbilical cord stump?	0=No<br>1=Yes<br>9=Don't Know	☐
**26.**	During the illness that led to death, did the baby have redness of the umbilical cord stump?	0=No (Go to Q27)<br>1=Yes (Go to Q26a)<br>9=Don't Know (Go to Q27)	☐
	**26a.** Did the redness of the cord stump extend onto the abdominal skin?	0=No<br>1=Yes<br>9=Don't Know	☐
**27.**	During the illness that led to death, did the baby have skin bumps containing pus or a single large area of pus?	0=No<br>1=Yes<br>9=Don't Know	☐
**28.**	During the illness that led to death, did the baby have ulcers (pits)?	0=No<br>1=Yes<br>9=Don't Know	☐
**29.**	During the illness that led to death, did the baby have an area of the skin with redness and swelling	0=No<br>1=Yes<br>9=Don't Know	☐
**30.**	During the illness that led to death, did the baby have and area of skin that turned black?	0=No<br>1=Yes<br>9=Don't Know	☐
**31.**	During the illness that led to death, did the baby bleed from anywhere?	0=No (Go to Q32)<br>1=Yes (Go to Q31a)<br>9=Don't Know (Go to Q32)	☐
	**31a.** From where did the baby bleed?	______________________________<br>______________________________	
**32.**	During the illness that led to death, did the baby have more frequent loose or liquid stools than usual?	0=No (Go to Q33)<br>1=Yes (Go to Q32a)<br>9=Don't Know (Go to Q33)	☐
	**32a.** How many stools did the baby have on the day that diarrhea/loose stools were most frequent?	01-98 = Number of stools<br>99=Don't Know	☐☐
**33.**	During the illness that led to death, did the baby vomit everything each time after feeding?	0=No<br>1=Yes<br>9=Don't Know	☐
**34.**	During the illness that led to death, did the baby have yellow skin?	0=No<br>1=Yes<br>9=Don't Know	☐

**35.**	During the illness that led to death, did the baby have yellow eyes?	0=No<br>1=Yes<br>9=Don't Know	☐
**36.**	Did the baby appear to be healthy and then just die suddenly?	0=No<br>1=Yes<br>9=Don't Know	☐
**37.**	Did the child receive any medical treatment before death?	0=No (Go to Section D)<br>1=Yes (Go to Q37a-b)<br>9=Don't Know (Section D)	☐
	**37a. First Treatment**		
	1. By whom …?	______________________	
	2. Where …?	______________________	
	3. What treatment ….?	______________________	
	**37b. Second Treatment**		
	1. By whom …?	______________________	
	2. Where …?	______________________	
	3. What treatment ….?	______________________	

Section D: Narratives

1. Please tell me in your own words how this baby died

____

____

____

____

____

____

____

____

____

____

2. Please describe the health of the mother in this pregnancy and tell me about the labor and delivery. Please also tell me about any problems experienced by the mother and whether she received care for these problems.
