# Supplementary material for: Causes of neonatal mortality using verbal autopsies in rural Southern Nepal, 2010–2017
Source: PLOS Glob Public Health. 2022 Sep 15;2(9):e0001072. doi: 10.1371/journal.pgph.0001072 (PMC10021801; doi:10.1371/journal.pgph.0001072)
Supplement: S1 Fig — (DOCX) [file pgph.0001072.s003.docx]

**S1 Fig:** Neonatal mortality rates from 2011 to 2016 in Nepal (The World Bank data^16^) and in the Sarlahi District (study data)
