# Supplementary material for: Causes of neonatal mortality using verbal autopsies in rural Southern Nepal, 2010–2017
Source: PLOS Glob Public Health. 2022 Sep 15;2(9):e0001072. doi: 10.1371/journal.pgph.0001072 (PMC10021801; doi:10.1371/journal.pgph.0001072)
Supplement: S2 Fig — (DOCX) [file pgph.0001072.s004.docx]

**S2 Fig**: Location of births (a, n=28,659) and neonatal deaths (b, n=984) from 2011 to 2017
