# Supplementary figures and images for: Causes of neonatal mortality using verbal autopsies in rural Southern Nepal, 2010–2017
Source: PLOS Glob Public Health. 2022 Sep 15;2(9):e0001072. doi: 10.1371/journal.pgph.0001072 (PMC10021801; doi:10.1371/journal.pgph.0001072)

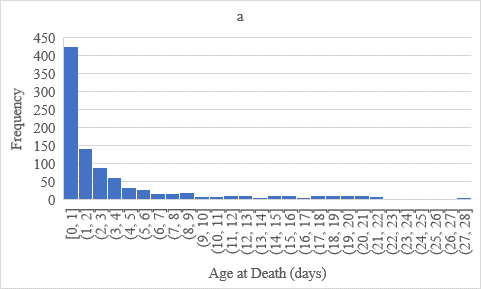


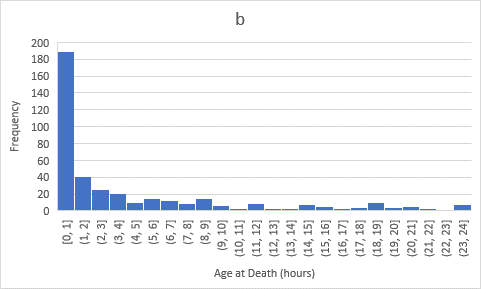


**S3 Fig**: Frequency of Neonatal Deaths in days (a) and within the first day in hours (b)

Supplement: S3 Fig — Frequency of Neonatal Deaths in days (a) and within the first day in hours (b). (DOCX) [file pgph.0001072.s005.docx]
