# Supplementary material for: Causes of neonatal mortality using verbal autopsies in rural Southern Nepal, 2010–2017
Source: PLOS Glob Public Health. 2022 Sep 15;2(9):e0001072. doi: 10.1371/journal.pgph.0001072 (PMC10021801; doi:10.1371/journal.pgph.0001072)
Supplement: S4 Fig — (DOCX) [file pgph.0001072.s006.docx]

**S4 Fig:** Symptoms Reported in Verbal Autopsies by Age at Death
