# Supplementary material for: Causes of neonatal mortality using verbal autopsies in rural Southern Nepal, 2010–2017
Source: PLOS Glob Public Health. 2022 Sep 15;2(9):e0001072. doi: 10.1371/journal.pgph.0001072 (PMC10021801; doi:10.1371/journal.pgph.0001072)
Supplement: S1 Table — (DOCX) [file pgph.0001072.s007.docx]

|  | | Neonatal Deaths | | | |
| --- | --- | --- | --- | --- | --- |
|  |  | All | <1 day | 1-7 days | 7-28 days |
| n | | 984 | 418 (42.5) | 380 (38.6) | 186 (18.9) |
| Preterm <34w0d | | 275 | 147 (53.5) | 93 (33.8) | 35 (12.7) |
| Preterm 34w0d- 36w6d | | 140 | 52 (37.1) | 52 (37.1) | 36 (25.7) |
| Sex (n, %) | Male | 537 | 225 (41.9) | 216 (40.2) | 96 (17.9) |
|  | Female | 443 | 189 (42.7) | 164 (37.0) | 90 (20.3) |
|  | Missing | 4 | 4 (100.0) | 0 (0) | 0 (0) |
| Multiples (n, %) | Singleton | 878 | 375 (42.7) | 336 (38.3) | 167 (19.0) |
|  | Twin | 99 | 42 (42.4) | 41 (41.4) | 16 (16.2) |
|  | Triplet | 7 | 1 (14.3) | 3 (42.9) | 3 (42.9) |

**S1 Table:** Demographic and clinical characteristics of neonatal deaths in Sarlahi, Nepal, 2010-2017
